# Enhancing Large Language Models for Improved Accuracy and Safety in Medical Question Answering: Comparative Study

**DOI:** 10.2196/70190

**Published:** 2025-12-02

**Authors:** Dingqiao Wang, Jinguo Ye, Jingni Li, Jiangbo Liang, Qikai Zhang, Qiuling Hu, Caineng Pan, Dongliang Wang, Zhong Liu, Wen Shi, Mengxiang Guo, Fei Li, Wei Du, Ying-Feng Zheng

**Affiliations:** 1 State Key Laboratory of Ophthalmology, Zhongshan Ophthalmic Center, Sun Yat-sen University, Guangdong Provincial Key Laboratory of Ophthalmology and Visual Science, Guangdong Provincial Clinical Research Center for Ocular Diseases Guangzhou China; 2 Department of Ophthalmology, Eighth Affiliated Hospital of Sun Yat-sen University Shenzhen, Guangdong China; 3 Guangzhou Women and Children's Medical Center Guangzhou China

**Keywords:** large language models, ChatGPT, medical question answering, health care communication, retrieval-augmented generation

## Abstract

**Background:**

Large language models (LLMs) offer the potential to improve virtual patient-physician communication and reduce health care professionals’ workload. However, limitations in accuracy, outdated knowledge, and safety issues restrict their effective use in real clinical settings. Addressing these challenges is crucial for making LLMs a reliable health care tool.

**Objective:**

This study aimed to evaluate the efficacy of Med-RISE, an information retrieval and augmentation tool, in comparison with baseline LLMs, focusing on enhancing accuracy and safety in medical question answering across diverse clinical domains.

**Methods:**

This comparative study introduces Med-RISE, an enhanced version of a retrieval-augmented generation framework specifically designed to improve question-answering performance across wide-ranging medical domains and diverse disciplines. Med-RISE consists of 4 key steps: query rewriting, information retrieval (providing local and real-time retrieval), summarization, and execution (a fact and safety filter before output). This study integrated Med-RISE with 4 LLMs (GPT-3.5, GPT-4, Vicuna-13B, and ChatGLM-6B) and assessed their performance on 4 multiple-choice medical question datasets: MedQA (US Medical Licensing Examination), PubMedQA (original and revised versions), MedMCQA, and EYE500. Primary outcome measures included answer accuracy and hallucination rates, with hallucinations categorized into factuality (inaccurate information) or faithfulness (inconsistency with instructions) types. This study was conducted between March 2024 and August 2024.

**Results:**

The integration of Med-RISE with each LLM led to a substantial increase in accuracy, with improvements ranging from 9.8% to 16.3% (mean 13%, SD 2.3%) across the 4 datasets. The enhanced accuracy rates were 16.3%, 12.9%, 13%, and 9.8% for GPT-3.5, GPT-4, Vicuna-13B, and ChatGLM-6B, respectively. In addition, Med-RISE effectively reduced hallucinations, with reductions ranging from 11.8% to 18% (mean 15.1%, SD 2.8%), factuality hallucinations decreasing by 13.5%, and faithfulness hallucinations decreasing by 5.8%. The hallucination rate reductions were 17.7%, 12.8%, 18%, and 11.8% for GPT-3.5, GPT-4, Vicuna-13B, and ChatGLM-6B, respectively.

**Conclusions:**

The Med-RISE framework significantly improves the accuracy and reduces the hallucinations of LLMs in medical question answering across benchmark datasets. By providing local and real-time information retrieval and fact and safety filtering, Med-RISE enhances the reliability and interpretability of LLMs in the medical domain, offering a promising tool for clinical practice and decision support.

## Introduction

Large language models (LLMs), such as ChatGPT, have emerged as a powerful paradigm in natural language processing [1]. With its humanlike conversational capabilities, ChatGPT has become a potent tool for medical question answering (QA), improving virtual patient-physician communication and reducing health care professionals’ workload [2-7].

Previous studies have focused on assessing the performance of LLMs in medical QA on standard datasets, such as MedMCQA, MedQA, and PubMedQA, and in specific specialties such as surgery, oncology, ophthalmology, and radiology [8,9]. The accuracy of models such as GPT-3.5 (50%-60%) and GPT-4 (70%-80%) is insufficient for clinical application, underscoring the need for further enhancements of LLMs regarding domain-specific medical knowledge [10-18]. Furthermore, the “hallucination” phenomenon in LLMs, which leads to factually inaccurate or irrelevant content, presents serious risks to patient care [19-23]. In medical practice, these hallucinations can lead to erroneous information, unsupported diagnoses, or inappropriate treatments. Consequently, many studies that have assessed LLM performance in medical applications highlight that major challenges in applying LLMs to clinical settings are insufficient accuracy; outdated knowledge; and potential safety issues, including hallucinations and bias [18,24,25]. Enhancing accuracy, timeliness, and safety is essential to make LLMs reliable for health care, ultimately improving patient outcomes.

Retrieval-augmented generation (RAG) is a promising strategy to enhance the accuracy of medical QA tasks and reduce hallucinations [26-29]. RAG improves LLMs’ responses by retrieving relevant documents from external knowledge, grounding the responses in factual information and increasing their reliability [30]. Previous studies have investigated the use of external knowledge to augment LLMs in medical domains, such as Almanac [31] and RECTIFIER [32]. However, most of these studies that have used retrieval techniques are based on small, predownloaded datasets, leading RAG models to be applied mostly in specific disciplines rather than broadly across medical fields [33-36]. In our previous study, we developed an RAG framework called RISE to improve LLMs’ performance in diabetes-related QA, achieving significant enhancements compared to the base LLMs [37]. Building on this framework, further exploration and refinement of knowledge augmentation techniques are warranted to expand its application across broader medical domains.

In this study, we introduced Med-RISE, an enhanced version of the RAG framework specifically designed to improve performance in medical QA tasks across broader medical domains. We assessed the impact of integrating the Med-RISE framework with the GPT-3.5, GPT-4, Vicuna-13B, and ChatGLM-6B LLMs and quantitatively evaluated its improvements in accuracy and reductions in hallucinations in the reasoning process using standard medical datasets, including MedQA (US Medical Licensing Examination [USMLE]), PubMedQA, and MedMCQA.

## Methods

### The Framework of Med-RISE

#### Overview

**Figure 1:**
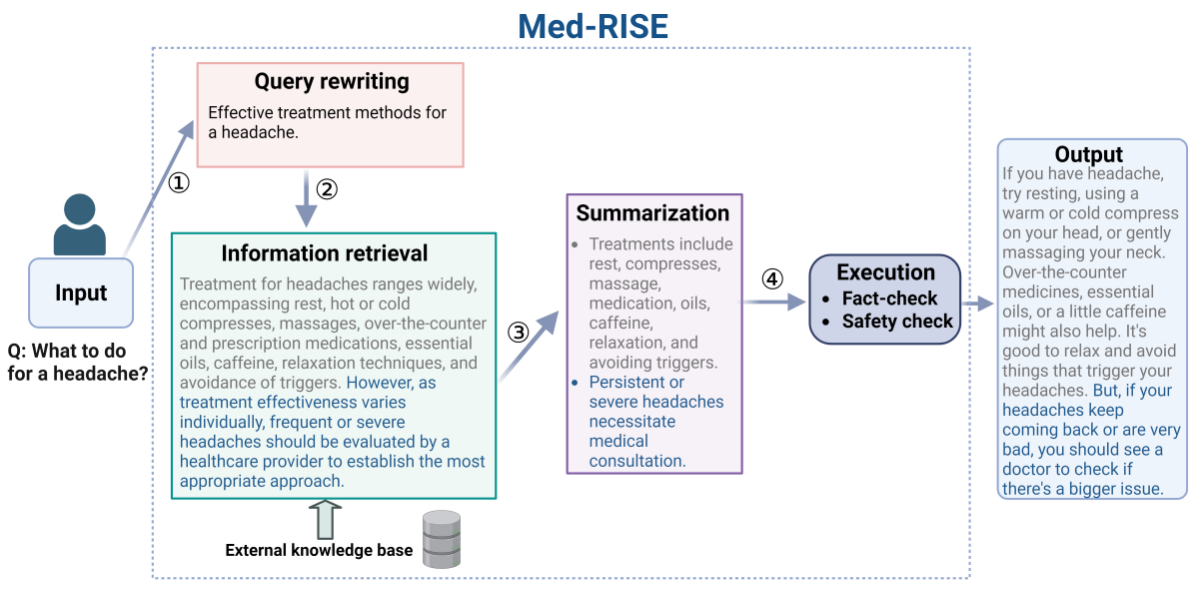
The Med-RISE framework: (1) the query rewriting step refines user queries using advanced large language models for better retrieval; (2) the information retrieval step searches for relevant context from an expanded medical local database and external real-time academic sources; (3) the summarization step summarizes the retrieved information into concise key points; and (4) the execution step generates the final answer, with fact-checking and safety validation to filter out incorrect, biased, or unsafe information before output, ensuring accuracy and safety.

In this study, we developed an advanced framework called Med-RISE to enhance the performance of LLMs in medical QA tasks. Building on our previous work on the RISE framework [37], which focused on diabetes-related QA in a single specialty domain, Med-RISE expands the retrieval database and incorporates more authoritative medical sources, making it suitable for diverse medical domains. The Med-RISE implementation requires 8 V100 graphics processing units (GPUs; 32 GB each) and 4 TB of storage for the medical database and achieves response times of 5 to 20 seconds using parallel retrieval strategies. The Med-RISE framework involves 4 key steps: query rewriting, information retrieval, summarization, and execution (Figure 1). The framework is implemented using the Python programming language (Python Software Foundation) on the Ubuntu operating system (Canonical Ltd). We provide open-source code (Multimedia Appendix 1) that supports both web deployment and mobile app integration.

#### Query Rewriting

In this step, Med-RISE first identifies the intent of the user or the patient’s query through the following prompt: “Please identify the intent of the patient’s query and select the most appropriate category from a list of predefined intents.” The 50 intent categories were developed by our team through analysis of clinical practice patterns and validated by clinical physicians (YZ). The LLM selects the single most appropriate intent category using natural language processing. The complete list of the 50 intent categories is provided in Multimedia Appendix 1.

Med-RISE uses LLMs such as GPT-3.5, GPT-4, Vicuna-13B, and ChatGLM-6B to refine the initial query. The refinement process involves addressing typographical errors, introducing related terms, expanding the range of possible matches, and enhancing overall retrieval precision. This step ensures that the rewritten query accurately captures the user’s intent and is well suited for subsequent information retrieval.

#### Information Retrieval

Med-RISE uses a dual retrieval approach combining local database and real-time academic sources to ensure comprehensive and current medical information. The system first converts the rewritten query into vector representation using the OpenAI Text-Embedding-ADA-002 model and then applies the Faiss similarity search algorithm to identify the 5 most relevant documents from the local database. Simultaneously, the framework performs automatic online retrieval through the Google search engine, filtering results using a predefined white list of 200 authoritative medical websites and academic institutions. This white list encompasses major medical journals (*The New England Journal of Medicine*, *Lancet*, and *The Journal of the American Medical Association*), medical databases (PubMed and Cochrane Library), professional medical organizations (the World Health Organization, Centers for Disease Control and Prevention, and National Institutes of Health), specialized medical societies, and official websites of medical schools and teaching hospitals. When local and real-time retrieval results are consistent, the information is integrated and synthesized. When discrepancies arise, priority is given to real-time retrieved information to ensure up-to-date medical knowledge. The filtered documents are then analyzed and integrated by the LLMs before proceeding to the summarization and execution steps.

To optimize retrieval latency, Med-RISE implements several strategies. For local database retrieval, the system uses 8 V100 GPUs (32 GB each) with parallel processing capabilities and optimized indexing methods such as IndexIVFFlat. For real-time retrieval, asynchronous parallel processing across multiple GPUs and network stability enhancements are used to minimize response delays, achieving response times of 5 to 20 seconds.

#### Summarization

In this step, Med-RISE summarizes the retrieved content into a clear and concise format, focusing on key points and eliminating redundancy. The model is prompted with the following: “You are an assistant skilled in organizing text. Summarize the following content clearly and briefly, keeping the important points and removing repeated information.” This step ensures that the content is well suited for generating an effective response.

#### Execution

The final step involves generating the final answer from the summarized information using the LLMs. Before sharing with the user, the answer undergoes fact-checking and safety validation to filter out any biased, unsafe, or incorrect information, ensuring that the response is accurate, neutral, and safe.

For fact-checking, the retrieved information is broken down into individual claims, which are then verified against external knowledge sources to confirm their accuracy using the following prompt: “As an AI medical assistant, your current task is to break down your last response in the conversation into independent claims. Do not include claims about opinions, or subjective personal experiences.” These claims are then verified against external knowledge sources to confirm their accuracy using the following prompt: “As an AI medical assistant, your current task is to fact-check the claims based on the external knowledge provided. You should label each claim as true or false. After that, output all the true claims without saying anything else. Now it is your turn to fact-check the claims based on the external knowledge.”

For safety validation, 24 predefined rules were developed based on medical ethics principles, patient safety requirements, and responsible artificial intelligence guidelines. The rules were tested in pilot experiments and then reviewed and refined by 2 clinical experts (MG and FL) before finalization. The model is given the following prompt: “The final response should comply with the following 24 guidelines.” These rules prevent specific medical diagnoses, treatment advice, and harmful generalizations and ensure that the responses recommend professional consultation when appropriate.

This 2-step process ensures that the final summarized content is filtered for safety and correctness before being presented to the user.

### Local Database

The local database for Med-RISE was constructed using 5 main sources: PubMed, StatPearls, 15 widely used medical textbooks, clinical practice guidelines, and Wikipedia. Specifically, PubMed provides 60,000 carefully selected high-quality biomedical articles (2015-2024; impact factor of ≥5; *Journal Citation Reports* quartiles 1-2; ≥50 citations) covering a wide range of biomedical research [37,38]. StatPearls serves as a clinical decision support tool, and we included more than 3000 publicly available StatPearls articles. The 15 medical textbooks are widely used in medical education and are the main references for USMLE preparation. Clinical practice guidelines from major medical societies, including the American College of Physicians and Chinese Medical Association, were included as main references for clinical decision support. Wikipedia, an open-source encyclopedia, provides general medical knowledge to support broader medical topics. These sources cover various medical disciplines, including anatomy, pharmacology, pathology, pediatrics, psychiatry, internal medicine, and gynecology (Multimedia Appendix 2).

The collected documents (from the 5 main sources) underwent preprocessing to eliminate unstructured or extraneous information before being segmented into smaller units. Embeddings for these segments were generated using the OpenAI Text-Embedding-ADA-002 model and then indexed using the Faiss similarity search algorithm for efficient retrieval. The process for generating embeddings and retrieving data follows a methodology similar to that of our previous RISE framework [37]. Due to the stable nature of foundational medical knowledge, the local database is updated annually through manual curation by our team according to our local database inclusion criteria.

After receiving a user’s query, the system transforms the rewritten query into an embedding vector. This vector is then matched against the database using cosine similarity to identify the 5 most relevant segments, which form the knowledge context for the query.

### Study Design

**Figure 2:**
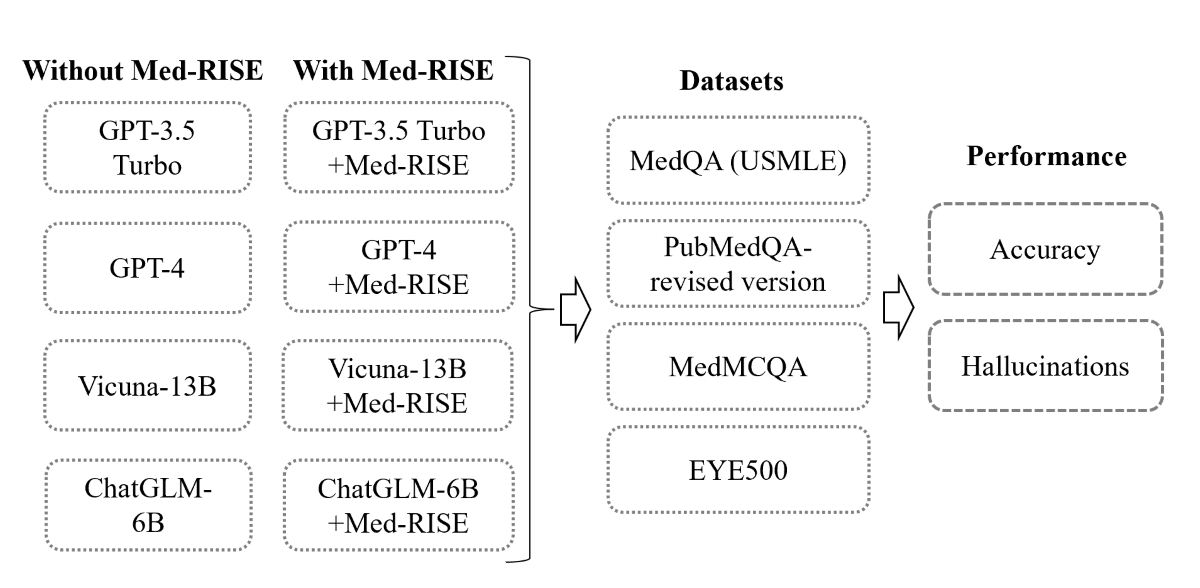
Flowchart of the overall study design. This study examined the accuracy and hallucination rates of large language models across 4 medical datasets with and without the Med-RISE framework. USMLE: US Medical Licensing Examination.

As shown in Figure 2, our study focused on assessing how the Med-RISE framework influences the effectiveness of LLMs in answering medical questions. We selected 4 representative LLMs: proprietary models GPT-3.5 and GPT-4 and open-source LLMs Vicuna-13B and ChatGLM-6B. These models were accessed and used between March 2024 and August 2024. These models have distinct characteristics in terms of performance, organization, source of training data, and parameter size (Table S1 in Multimedia Appendix 3). By testing Med-RISE on these diverse LLMs, we aimed to demonstrate the effectiveness and versatility of the Med-RISE framework.

### Ethical Considerations

The Zhongshan Ophthalmic Centre ethics committee (Guangzhou, China) approved this study (2024KYPJ124). As this research did not involve the collection of patient information or data, the ethics approval document includes an exemption from informed consent.

### Validation Assessment

In the accuracy evaluation, we compared the performance of various LLMs with and without the application of the Med-RISE framework. This comparison was conducted on multiple-choice questions derived from several datasets: MedQA (USMLE), PubMedQA, MedMCQA, and EYE500. Accuracy was defined as the proportion of questions answered correctly out of the total number of questions compared to the standard answers provided in each dataset.

We assessed the presence of hallucinations in the reasoning process of the LLMs using the chain-of-thought (CoT) approach. Hallucinations are defined as content that is either factually incorrect or unrelated to the given information and are categorized into 2 main types: factuality hallucinations and faithfulness hallucinations [39]. Factuality hallucinations occur when the generated content conflicts with real-world information, which includes factual inconsistency (contradicting real-world information) and factual fabrication (generating content that cannot be verified using real-world information). Faithfulness hallucinations involve the inconsistency between the generated content and user instructions, input context, or internal logical coherence, which includes instruction inconsistency (output deviating from user instructions), context inconsistency (output contradicting contextual information), and logical inconsistency (inconsistency between reasoning steps and the final answer). The hallucination rate refers to the percentage of generated responses that included any form of hallucination. Two clinical physicians (DW and WS) independently evaluated the presence of each specific hallucination type, with disagreements resolved through consensus by a senior physician (YZ). Furthermore, we evaluated the proportion of each specific hallucination type.

### Medical Datasets Used

In this study, we evaluated the performance of the Med-RISE framework using 4 medical datasets: MedQA (USMLE) [40], MedMCQA [41], PubMedQA [42], and EYE500 (Table S2 in Multimedia Appendix 3). MedQA (USMLE), MedMCQA, and EYE500 are multiple-choice medical board exam question datasets, whereas PubMedQA is a medical reading comprehension dataset [21,43]. For each dataset, we randomly selected 200 questions to reduce computational costs while maintaining evaluation comprehensiveness as preliminary testing showed comparable accuracy rates between the full datasets and subsampled questions.

The MedQA (USMLE) dataset, sourced from the USMLE, consists of challenging multiple-choice questions designed to assess medical knowledge. The MedMCQA dataset consists of diverse multiple-choice questions from the All India Institute of Medical Science and National Eligibility cum Entrance Test (Postgraduate) exam, encompassing various health care topics and medical subjects. These datasets cover diverse medical disciplines, including clinical medicine (internal medicine, surgery, pediatrics, and psychiatry), basic medical sciences (anatomy, physiology, and pathology), and specialized fields. We also introduce the EYE500 dataset, explicitly developed for this study in collaboration with ophthalmologists from Zhongshan Ophthalmic Center, Sun Yat-sen University (Multimedia Appendix 4).

The PubMedQA dataset, a medical reading comprehension dataset, contains questions based on biomedical article abstracts from the PubMed database, requiring models to answer questions with “yes,” “no,” or “maybe” based on the provided abstract information. However, the accuracy of the answers may be affected by the quality and interpretation of the original studies. To ensure the reliability of the 200 questions and answers used in this study, we had 2 medical experts (DW and WS) independently reverify them. In cases of disagreement, a senior physician (YZ) made the final decision, creating the PubMedQA—revised version (Multimedia Appendix 5).

### Prompts

We used 2 prompting strategies: zero-shot prompting for accuracy assessment (eg, “Please provide the answer to this question”) and CoT prompting for hallucination evaluation to obtain step-by-step reasoning processes (eg, “Please use step-by-step reasoning to analyze this medical question systematically”) [43].

### Statistical Analysis

The data analysis was conducted using the SPSS software (version 25.0; IBM Corp). Chi-square tests were used to compare accuracy and hallucination rates. Two clinicians assessed hallucinations (>5 years of experience each), with disagreements resolved by a senior physician. Statistical analysis was conducted in consultation with Jin Ling, a professional biostatistician from Zhongshan Ophthalmic Center. Statistical significance was set at a *P* value of <.05.

## Results

### Enhanced Accuracy of LLMs Across All Datasets With Med-RISE Integration

#### Overview

**Figure 3:**
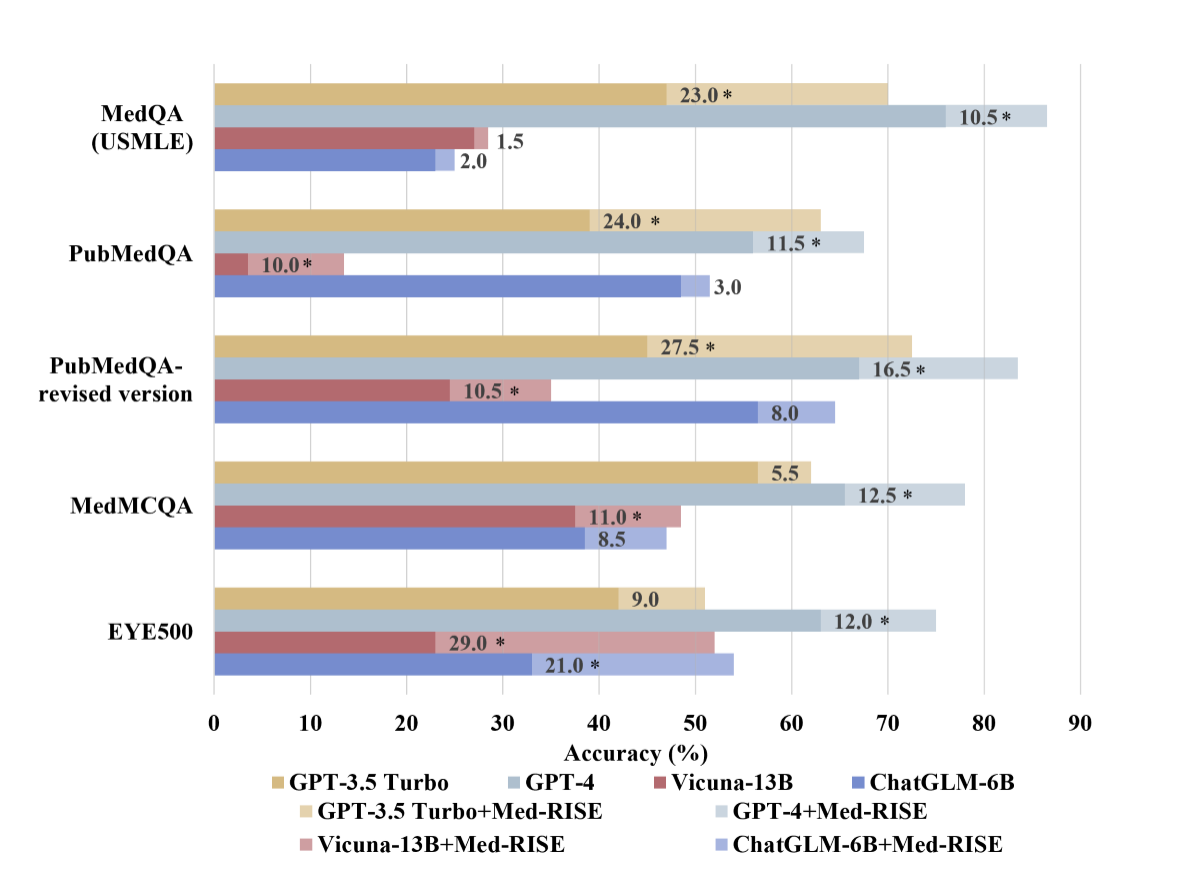
Bar plot of enhanced accuracy in the large language models with Med-RISE. Each bar shows the accuracy of each model on a specific dataset, with the numbers representing the difference in accuracy before and after Med-RISE integration. *P<.05. USMLE: US Medical Licensing Examination.

This section presents a comparative analysis of the performance of the 4 LLMs—GPT-3.5, GPT-4, Vicuna-13B, and ChatGLM-6B—on the MedQA (USMLE), PubMedQA, PubMedQA—revised version, MedMCQA, and EYE500 medical datasets. The integration of Med-RISE with each of these LLMs significantly improved accuracy (Table 1 and Figure 3).

The incorporation of Med-RISE into each LLM led to a substantial increase in accuracy across the 4 datasets. Med-RISE enhanced overall accuracy rates, with improvements ranging from 9.8% to 16.3% (mean 13%, SD 2.3%) across the 4 models. Specifically, GPT-3.5 accuracy improved from 47.6% to 63.9% (improvement of 16.3%), GPT-4 accuracy improved from 67.9% to 80.8% (improvement of 12.9%), Vicuna-13B accuracy improved from 28% to 41% (improvement of 13%), and ChatGLM-6B accuracy improved from 37.8% to 47.6% (improvement of 9.8%).

**Table 1:** Accuracy of the large language models on biomedical question answering datasets.^a^

Dataset	GPT-3.5 Turbo, n (%)				GPT-4, n (%)				Vicuna-13B, n (%)				ChatGLM-6B, n (%)		
	Without Med-RISE	With Med-RISE	*P* value	Without Med-RISE		With Med-RISE	*P* value	Without Med-RISE		With Med-RISE	*P* value	Without Med-RISE		With Med-RISE	*P* value
MedQA (USMLE^b^)	94 (47.0)	140 (70.0)	<.001	152 (76.0)		173 (86.5)	.02	54 (27.0)		57 (28.5)	.74	46 (23.0)		50 (25.0)	.64
PubMedQA	78 (39.0)	126 (63.0)	<.001	112 (56.0)		135 (67.5)	.02	7 (3.5)		27 (13.5)	.001	97 (48.5)		103 (51.5)	.55
PubMedQA—revised version	90 (45.0)	145 (72.5)	<.001	134 (67.0)		167 (83.5)	<.001	49 (24.5)		70 (35.0)	.02	113 (56.5)		129 (64.5)	.10
MedMCQA	113 (56.5)	124 (62.0)	.26	131 (65.5)		156 (78.0)	.01	75 (37.5)		97 (48.5)	.03	77 (38.5)		94 (47.0)	.09
EYE500	84 (42.0)	102 (51.0)	.07	126 (63.0)		150 (75.0)	.009	46 (23.0)		104 (52.0)	<.001	66 (33.0)		108 (54.0)	<.001
Total^c^	381 (47.6)	511 (63.9)	.001	543 (67.9)		646 (80.8)	.01	224 (28.0)		328 (41.0)	.02	302 (37.8)		381 (47.6)	.04

^a^The *P* values correspond to the chi-square tests comparing each language model’s accuracy rate with and without Med-RISE.

^b^USMLE: US Medical Licensing Examination.

**^c^**Represents the combined results from 4 datasets (MedQA, PubMedQA—revised version, MedMCQA, and EYE500) with 200 questions each (n=800).

#### GPT-3.5

The integration of Med-RISE led to an accuracy increase from 47% to 70% (a 23% improvement; *P*<.001) in MedQA (USMLE), from 39% to 63% (a 24% improvement; *P*<.001) in PubMedQA, from 45% to 72.5% (a 27.5% improvement; *P*<.001) in PubMedQA—revised version, from 56.5% to 62% (a 5.5% improvement; *P*=.26) in MedMCQA, and from 42% to 51% (a 9% improvement; *P*=.07) in EYE500.

#### GPT-4

After incorporating Med-RISE, GPT-4 significantly improved in accuracy across all datasets. The accuracy on the MedQA (USMLE) dataset increased from 76% to 86.5% (a 10.5% improvement; *P*=.02). For PubMedQA, accuracy increased from 56% to 67.5% (an 11.5% improvement; *P*=.02). For PubMedQA—revised version, accuracy increased from 67% to 83.5% (a 16.5% improvement; *P*<.001). For MedMCQA, accuracy increased from 65.5% to 78% (a 12.5% improvement; *P*=.01). For EYE500, there was a substantial increase in accuracy from 63% to 75% (a 12% improvement; *P*=.009).

#### Vicuna-13B

With the application of Med-RISE, Vicuna-13B improved in accuracy from 27% to 28.5% (a 1.5% improvement; *P*=.74) in MedQA (USMLE), from 3.5% to 13.5% (a 10% improvement; *P*=.001) in PubMedQA, from 24.5% to 35% (a 10.5% improvement; *P*=.02) in PubMedQA—revised version, from 37.5% to 48.5% (an 11% improvement; *P*=.03) in MedMCQA, and from 23% to 52% (a 29% improvement; *P*<.001) in EYE500.

#### ChatGLM-6B

The implementation of Med-RISE with ChatGLM-6B resulted in an increase in accuracy from 23% to 25% (a 2% improvement; *P*=.64) in MedQA (USMLE), from 48.5% to 51.5% (a 3% improvement; *P*=.55) in PubMedQA, from 56.5% to 64.5% (an 8% improvement; *P*=.10) in PubMedQA—revised version, from 38.5% to 47% (an 8.5% improvement; *P*=.09) in MedMCQA, and from 33% to 54% (a 21% improvement; *P*<.001) in EYE500.

### Assessing Hallucinations in the Reasoning Process of LLMs via CoT

The reasoning process of the LLMs was evaluated using the CoT method, specifically focusing on the tendency of these models to generate hallucinations. It was observed that all LLMs were prone to this issue, albeit to varying extents. The average hallucination rates were 78.6% for Vicuna-13B, 67.3% for ChatGLM-6B, and 56.3% for GPT-3.5, with the lowest being that for GPT-4 at 35.3% (Table 2).

**Figure 4:**
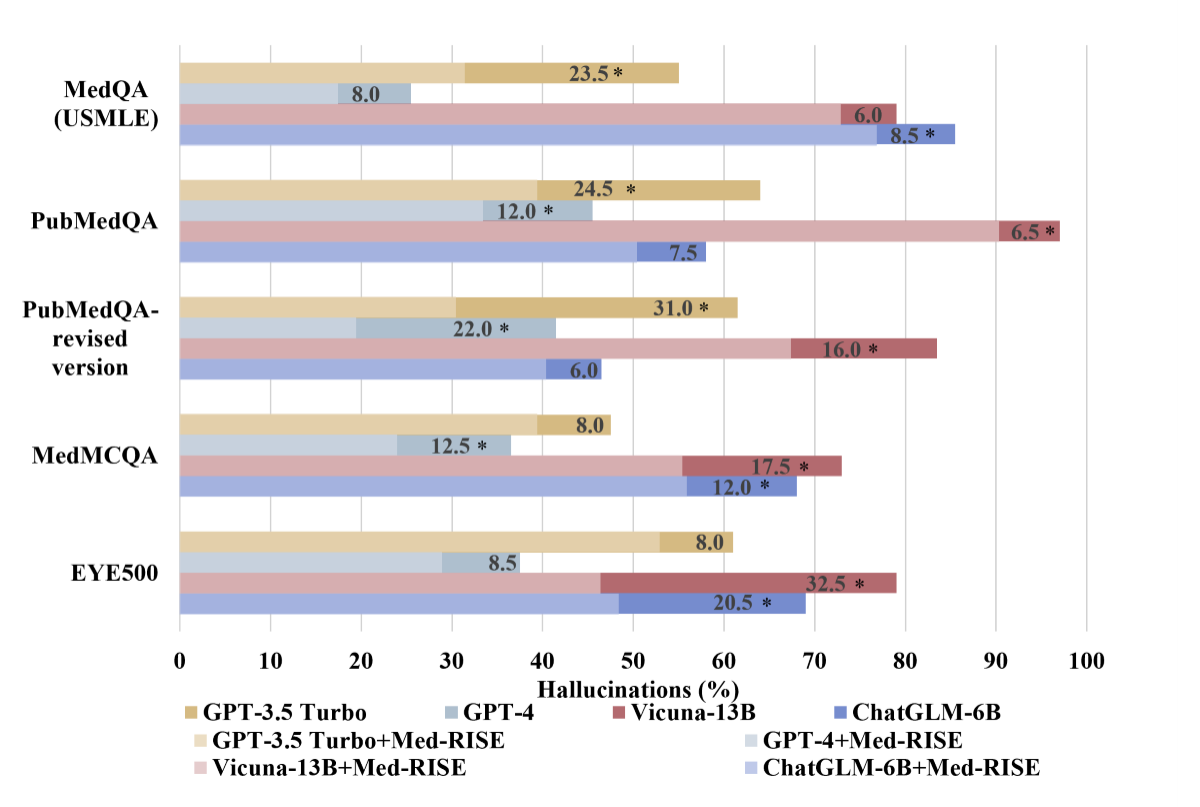
Bar plot of hallucination reduction in the large language models with Med-RISE. Each bar shows the hallucination rate of each model, with the numbers representing the difference in hallucination rate before and after Med-RISE integration. *P<.05. USMLE: US Medical Licensing Examination.

Integrating Med-RISE into these LLMs substantially reduced hallucination occurrences, with reductions ranging from 11.8% to 18% (mean 15.1%, SD 2.8%) across 4 datasets: MedQA (USMLE), PubMedQA—revised version, MedMCQA, and EYE500 (Figure 4). Specifically, GPT-3.5 hallucination rates decreased from 56.3% to 38.6% (reduction of 17.7%), GPT-4 hallucination rates decreased from 35.3% to 22.5% (reduction of 12.8%), Vicuna-13B hallucination rates decreased from 78.6% to 60.6% (reduction of 18%), and ChatGLM-6B hallucination rates decreased from 67.3% to 55.5% (reduction of 11.8%). Furthermore, the application of Med-RISE to the LLMs even led to a reduction in hallucinations exceeding 20% in certain cases. In the MedQA (USMLE) dataset, GPT-3.5 exhibited a significant reduction in hallucinations of 23.5% (*P*<.001) after Med-RISE integration. In the EYE500 dataset, Vicuna-13B and ChatGLM-6B exhibited significant reductions in hallucinations of 32.5% (*P*<.001) and 20.5% (*P*<.001), respectively, after Med-RISE integration. Similarly, the PubMedQA—revised version revealed a substantial decrease in hallucinations of 31% (*P*<.001) for GPT-3.5 and 22% (*P*<.001) for GPT-4 when applying Med-RISE.

**Table 2:** Incidence of hallucinations in chain of thought among the large language models.^a^

Dataset	GPT-3.5 Turbo, n (%)				GPT-4, n (%)				Vicuna-13B, n (%)				ChatGLM-6B, n (%)		
	Without Med-RISE	With Med-RISE	*P* value	Without Med-RISE		With Med-RISE	*P* value	Without Med-RISE		With Med-RISE	*P* value	Without Med-RISE		With Med-RISE	*P* value
MedQA (USMLE^b^)	110 (55.0)	63 (31.5)	<.001	51 (25.5)		35 (17.5)	.05	158 (79.0)		146 (73.0)	.16	171 (85.5)		154 (77.0)	.03
PubMedQA	128 (64.0)	79 (39.5)	<.001	91 (45.5)		67 (33.5)	.01	194 (97.0)		181 (90.5)	.007	116 (58.0)		101 (50.5)	.13
PubMedQA—revised version	123 (61.5)	61 (30.5)	<.001	83 (41.5)		39 (19.5)	<.001	167 (83.5)		135 (67.5)	<.001	93 (46.5)		81 (40.5)	.27
MedMCQA	95 (47.5)	79 (39.5)	.11	73 (36.5)		48 (24.0)	.007	146 (73.0)		111 (55.5)	<.001	136 (68.0)		112 (56.0)	.01
EYE500	122 (61.0)	106 (53.0)	.11	75 (37.5)		58 (29.0)	.07	158 (79.0)		93 (46.5)	<.001	138 (69.0)		97 (48.5)	<.001
Total^c^	450 (56.3)	309 (38.6)	.009	282 (35.3)		180 (22.5)	.003	629 (78.6)		485 (60.6)	<.001	538 (67.3)		444 (55.5)	.02

^a^The *P* values correspond to the chi-square tests comparing each language model’s hallucination rate with and without Med-RISE integration.

^b^USMLE: US Medical Licensing Examination.

**^c^**Represents the combined results from 4 datasets (MedQA, PubMedQA—revised version, MedMCQA, and EYE500) with 200 questions each (n=800).

### Reduction in Different Hallucination Types Through Med-RISE

Table 3 presents the proportion of different types of hallucinations observed in CoT from the 4 different LLMs with and without Med-RISE integration. For this analysis, 50 questions were randomly selected from the 4 datasets, and these questions were answered by the 4 models with and without Med-RISE, resulting in 200 question-answer pairs for each set. The hallucinations were categorized into 2 main types: factuality hallucinations and faithfulness hallucinations, with further subtypes for each.

The results showed that the application of Med-RISE led to reductions in both main categories of hallucinations, with factuality hallucinations decreasing by 13.5% and faithfulness hallucinations decreasing by 5.8%. When examining specific subtypes, factuality hallucinations showed reductions in both factual inconsistency (42/200, 21%) and factual fabrication (12/200, 6%). Similarly, faithfulness hallucinations demonstrated reductions across instruction inconsistency (8/200, 4%), context inconsistency (15/200, 7.5%), and logical inconsistency (12/200, 6%). Representative examples of these different hallucination types are shown in Textboxes S1 and S2 in Multimedia Appendix 3.

**Table 3:** Proportion of different types of hallucinations of the large language models in chain of thought (N=200)^a^.

Type of hallucination and pattern			Without Med-RISE, n (%)		With Med-RISE, n (%)		Change, n (%)
**Factuality hallucination**							
	Factual inconsistency	85 (42.5)		43 (21.5)		42 (21.0)	
	Factual fabrication	38 (19.0)		26 (13.0)		12 (6.0)	
**Faithfulness hallucination**							
	Instruction inconsistency	23 (11.5)		15 (7.5)		8 (4.0)	
	Context inconsistency	29 (14.5)		14 (7.0)		15 (7.5)	
	Logical inconsistency	47 (23.5)		35 (17.5)		12 (6.0)	

^a^A total of 50 questions were randomly chosen from the 4 datasets. Each group had 200 question-answer pairs.

## Discussion

### Principal Findings

This study demonstrates the effectiveness of Med-RISE across multiple medical disciplines in enhancing the medical QA capabilities of LLMs. Our evaluation shows that Med-RISE achieved accuracy improvements ranging from 9.8% to 16.3% (mean 13%, SD 2.3%) across all tested LLMs and datasets while significantly reducing hallucinations, with reductions ranging from 11.8% to 18% (mean 15.1%, SD 2.8%), factuality hallucinations decreasing by 13.5%, and faithfulness hallucinations decreasing by 5.8%. These comprehensive improvements in both accuracy and hallucination mitigation demonstrate Med-RISE’s capability in addressing 2 fundamental challenges of medical LLMs: knowledge and response reliability.

### Comparison With Prior Work

Compared to previous RAG frameworks [44,45], which often depend on static and more limited knowledge bases [46,47], Med-RISE represents a significant advancement through its dynamic, real-time knowledge retrieval across multiple medical disciplines. Building on RISE [37], which we previously implemented for diabetes-specific QA, Med-RISE extends these capabilities across broader medical domains with enhanced safety verification. Previous approaches—such as the focus on diffuse large B-cell lymphoma by Soong et al [35] using 500 PubMed articles, the liver disease–specific LiVersa by Ge et al [34], the Almanac framework by Zakka et al [31] for treatment guidelines, and the medical textbook augmentation by Wang et al [26]—have demonstrated limited application as they were constrained by their fixed knowledge bases. More importantly, even with RAG implementation, LLMs still face inherent challenges regarding errors and hallucinations. Med-RISE uniquely addresses this through its additional layer of accuracy and safety verification before output generation, significantly reducing error rates in medical responses. This dual-stage approach—combining dynamic knowledge retrieval with accuracy and safety verification—makes Med-RISE a more reliable and generalizable tool for medical QA than conventional RAG frameworks.

### Research Implications

The integration of Med-RISE with LLMs achieved 2 critical improvements in medical QA performance: average accuracy increases of 13% across 4 general medical datasets and a 15% reduction in hallucinations, with the most substantial improvement in factual inconsistency (42/200, 21% reduction). These significant improvements stem from Med-RISE’s novel mechanism that combines real-time medical knowledge retrieval with accuracy and safety verification filters, effectively reducing both outdated information and factual errors. Med-RISE functions as an external augmentation framework that enhances domain-specific performance without modifying underlying model parameters, offering cost-effective adaptation compared to expensive model retraining while enabling broader institutional adoption. In medical applications, even small improvements in accuracy can have profound implications given the field’s sensitivity to precision. Each percentage point of enhanced accuracy directly impacts the quality of diagnoses, treatment strategies, and patient education. Medical misinformation can lead to severe consequences, from compromised clinical decision-making to patient safety risks [48]. Therefore, Med-RISE’s substantial improvements in both accuracy and reliability represent crucial advancements for deploying LLMs in health care settings.

### Future Directions

Med-RISE demonstrates significant potential for advancing clinical practice through its robust framework for medical applications. Its effectiveness stems from 2 key components: real-time knowledge retrieval across medical disciplines enabling transparent responses and verification of accuracy and safety. This proven performance allows for Med-RISE’s integration across various health care processes—from preconsultation screening and surgical consent to patient decision support, postoperative follow-up, disease consultation, and public health education. Our study also revealed that, while Med-RISE achieved improvements across all tested models, smaller-parameter models exhibited greater performance variability across different datasets, whereas larger models demonstrated more consistent gains. Future work may consider using larger models as the foundational framework to achieve more stable performance. Currently, Med-RISE achieves response times of 5 to 20 seconds through parallel retrieval processing (using 8 V100 GPUs of 32 GB each). For faster responses, performance can be enhanced through higher-performance GPUs, increased parallel processing, and improved network speeds.

### Limitations

This study has several limitations. It adopted random subsets from each dataset, which may lead to performance variations and might not fully reflect the outcomes of a comprehensive analysis of the entire dataset. Furthermore, Med-RISE’s impact on response time, computational requirements, and user experience remains to be explored. Future work should focus on expanding knowledge diversity and incorporating reinforcement learning methods such as proximal policy optimization [49] and direct preference optimization [50] to enhance Med-RISE’s alignment with human values and preferences, further improving response quality. Comprehensive evaluations that include feedback from clinical users and health care professionals in real clinical settings will also be essential to validate the framework’s effectiveness and refine its practical implementation.

### Conclusions

In conclusion, Med-RISE enhances the clinical application of LLMs through its unique integration of real-time knowledge retrieval and safety verification mechanisms. By significantly improving accuracy, reducing hallucinations, and enabling transparent responses through knowledge-grounded generation, the framework ensures safer and more reliable medical information delivery across diverse medical scenarios. Med-RISE demonstrates strong potential for advancing health care applications from patient consultation to public health education while maintaining essential safety standards.

## Appendix

Multimedia Appendix 1 Code for the Med-RISE framework.

Multimedia Appendix 2 Sample of Med-RISE local dataset (n=500).

Multimedia Appendix 3 Supplementary tables and figures.

Multimedia Appendix 4 Sample of EYE500 dataset (n=50).

Multimedia Appendix 5 PubMedQA_revised version (n=200).

## Abbreviations

CoT: chain-of-thought
GPU: graphics processing unit
LLM: large language model
QA: question answering
RAG: retrieval-augmented generation
USMLE: United States Medical Licensing Examination

## Data Availability

The code for the retrieval component of the Med-RISE framework is publicly available (Multimedia Appendix 1). A sample of the Med-RISE local database (n=500) is included in Multimedia Appendix 2. A subset of the EYE500 dataset (n=50) is provided in Multimedia Appendix 4. The revised PubMedQA dataset (PubMedQA—revised version; n=200) used in this study is available with open access in Multimedia Appendix 5. The full code and local database of Med-RISE in this study are available from the corresponding author on reasonable request.
