# Supplementary material for: Enhancing Large Language Models for Improved Accuracy and Safety in Medical Question Answering: Comparative Study
Source: JMIR Med Educ. 2025 Dec 2;11:e70190. doi: 10.2196/70190 (PMC12709156; doi:10.2196/70190)
Supplement: Multimedia Appendix 3 [file mededu_v11i1e70190_app3.docx]

**Supplemental Materials**

[Table S1. Overview of Large Language Models used in the study. 2](#_Toc184673027)

[Table S2. Summary of the medical question- answering datasets used in the study. 3](#_Toc184673028)

[Figure S1. Example of hallucination types in Chain of Thought (CoT) reasoning in each Large Language Model (Sample 1). 4](#_Toc184673029)

[Figure S2. Example of hallucination types in Chain of Thought (CoT) reasoning in each Large Language Model (Sample 2). 8](#_Toc184673030)

# Table S1. Overview of Large Language Models used in the study.

|  | GPT-3.5 Turbo | GPT-4 Turbo | Vicuna-13B | ChatGLM-6B |
| --- | --- | --- | --- | --- |
| Parameters | 175 billion | - | 13B | 6B |
| Training | Self-supervised learning | Self-supervised learning | Self-supervised pretraining | Self-supervised pretraining |
| Max Token Span | 4,096 tokens | 8,192 tokens | 2048 tokens | 13052 tokens |
| Training Cutoff | Sep 2021 | Sep 2021 | April 2023 | May 2023 |

^a^ Model Call Dates: March 2024 - August 2024.

# Table S2. Summary of the medical question- answering datasets used in the study.

|  | MedQA (USMLE) | PubMedQA | MedMCQA | EYE500 |
| --- | --- | --- | --- | --- |
| Format | Q+A  (4-5 Choices) | Q+ Context +A  (Yes/No/Maybe) | Q+A  (4 Choices and Explanations) | Q+A  (4 Choices) |
| Size | 12.7K | 1K | 194K | 0.5K |
| Domain | General medical knowledge in US medical licensing exam | Biomedical scientific articles | General medical knowledge in Indian medical entrance exams | Ophthalmological medical knowledge for residency training |
| Source (questions) | The United States Medical Licensing Examination (Step 1 and Step 2) | Expert-annotated PubMed abstracts | AIIMS and NEET PG entrance exams | Created by ophthalmology experts at Zhongshan Ophthalmic Center |

^a^ For each dataset, we randomly selected 200 questions to evaluate the performance of LLMs.

^b^ EYE500: Created by a collaborative team of ophthalmologists at Zhongshan Ophthalmic Center, Sun Yat-sen University, including 2 senior ophthalmologists and 2 residents. Questions cover core topics in ophthalmology residency training.

^c^ USMLE: the United States Medical Licensing Examination; AIIMS: India Institute of Medical Sciences; NEET PG: National Eligibility cum Entrance Test for Postgraduate.

# Figure S1. Example of hallucination types in Chain of Thought (CoT) reasoning in each Large Language Model (Sample 1).

| **MedQA (USMLE) [test: 772]:** A 25-year-old man presents to his primary care provider complaining of several weeks of intense itching, particularly localized around his anus. Of note, the patient denies any recent travel, sexual contacts, fever, dysuria, or diarrhea. His temperature is 98.1°F (36.7°C), blood pressure is 110/70 mmHg, pulse is 65/min, and respirations are 12/min. On exam, the patient demonstrates severe excoriations in his perianal region with slight erythema over the areas that are torn. He does not have any other dermatologic findings. The scotch tape test is positive. The patient has a previous documented allergy to albendazole. Which of the following is an alternative medication that can be used?  A) Amphotericin; B) Diethylcarbamazine; C) Ivermectin; D) Praziquantel; **E) Pyrantel pamoate.**  **GPT-Turbo 3.5**  The patient presents with localized intense itching around the anus, severe excoriations, and slight erythema in the perianal region. A positive scotch tape test suggests enterobiasis, also known as pinworm infection. Allergy to albendazole eliminates it as a treatment option. Let's review the alternative medications:  A) Amphotericin: It is an antifungal medication and ineffective against pinworm infection. Not the correct choice.  B) Diethylcarbamazine: It is primarily used for lymphatic filariasis and other parasitic infections, not for enterobiasis. Not the correct choice.  C) Ivermectin: It is a broad-spectrum antiparasitic medication effective against various parasites, including Enterobius vermicularis. Safe and suitable for enterobiasis treatment. Possible correct choice.  D) Praziquantel: It is used for cestode and trematode infections, not for pinworm infection. Not the correct choice.  E) Pyrantel pamoate: It is an anthelmintic medication commonly used for enterobiasis. Effective against Enterobius vermicularis. Another possible correct choice.  Considering the patient's allergy to albendazole, both Ivermectin and Pyrantel pamoate are suitable alternative medications for the treatment of enterobiasis. The final choice depends on factors such as availability, cost, and individual patient considerations. Consultation with a healthcare provider is recommended to determine the most appropriate alternative medication in this case.  **GPT-Turbo 3.5 + Med-RISE**  Based on the patient's symptoms and physical exam findings, it is likely that he has a parasitic infection, specifically a pinworm infection (Enterobius vermicularis). The positive scotch tape test, which involves placing a piece of clear tape against the perianal region and examining it under a microscope for pinworm eggs, supports this diagnosis.  The treatment of choice for pinworm infection is albendazole. However, since the patient has a documented allergy to albendazole, an alternative medication is needed.  Among the options provided, the most appropriate alternative medication for pinworm infection is Pyrantel pamoate (Option E). Pyrantel pamoate is an anthelmintic medication that is commonly used to treat pinworm infections. It works by paralyzing the worms, allowing them to be expelled from the body through bowel movements.  Therefore, the correct answer is E) Pyrantel pamoate.  **GPT-4**  The patient's symptoms and positive scotch tape test suggest a pinworm infection. Given his allergy to albendazole, an alternative treatment from the list provided is Pyrantel pamoate. This medication works effectively against pinworms by paralyzing them, and it is safe for use in cases where albendazole cannot be administered.  The correct answer is:  E) Pyrantel pamoate.  **GPT-4 + Med-RISE**  The clinical presentation of the patient described in this vignette is consistent with a pinworm infection (Enterobius vermicularis), which is characterized by intense perianal itching, particularly at night. The diagnosis is confirmed by the positive scotch tape test, which shows eggs of the parasite.  The first-line treatment for pinworm infection is typically albendazole or mebendazole. However, the patient has a documented allergy to albendazole, necessitating an alternative treatment.  Of the options listed:  Amphotericin B is an antifungal medication, not appropriate for treating parasitic worm infections.  Diethylcarbamazine is used for the treatment of certain filarial worm infections, not pinworms.  Ivermectin is a broad-spectrum antiparasitic used in the treatment of a variety of worm infestations and is effective against pinworms.  Praziquantel is used primarily for schistosomiasis and other fluke infections, not for pinworms.  Pyrantel pamoate is another antiparasitic that is effective against pinworms and could be used as an alternative in the case of albendazole allergy.  Considering the patient's allergy to albendazole, the best alternative medication from the list is:  E) Pyrantel pamoate. This medication causes paralysis of the parasites by acting as a neuromuscular blocking agent. It is safe and effective for the treatment of pinworm infections, making it a suitable alternative for patients who cannot take albendazole or mebendazole.  **Vicuna-13B**  Step 1: Symptoms  The patient presents with intense itching, particularly localized around his anus, with no recent travel, sexual contacts, fever, dysuria, or diarrhea.  Step 2: Medical history  The patient has a previous documented allergy to albendazole.  Step 3: Examination  On exam, the patient demonstrates severe excoriations in his perianal region with slight erythema over the areas that are torn. The scotch tape test is positive.  Step 4: Differential diagnosis  Based on the patient’s symptoms, medical history, and examination findings, the most likely diagnosis is a cutaneous larva migrans (CLM) caused by a filarial parasite.  Step 5: Treatment  The treatment of choice for CLM is ivermectin.  Therefore, the answer is C) Ivermectin.  **Vicuna-13B + Med-RISE**  Step 1: Observation and description of symptoms  The patient presents with intense perianal itching, particularly localized around his anus for several weeks. This is the main symptom that led to the suspicion of a parasitic skin infection.  Step 2: Differential diagnosis  Given the symptoms described, a possible differential diagnosis could be pinworm infection (Enterobius vermicularis) which is characterized by intense perianal itching, particularly at night. The diagnosis is confirmed by the positive scotch tape test, which shows eggs of the parasite.  Step 3: First-line treatment options  The first-line treatment for pinworm infection is typically albendazole or mebendazole. However, since the patient has a documented allergy to albendazole, an alternative treatment option must be considered.  Step 4: Alternative treatment options  Given the patient's history of an allergy to albendazole, and the positive scotch tape test for a parasitic skin infection, an alternative medication that can be used is option E) Pyrantel pamoate. This drug is safe and effective for the treatment of pinworm infections and causes paralysis of the parasites by acting as a neuromuscular blocking agent.  **ChatGLM-6B**   1. The patient is presenting with severe excoriations in his perianal region, which suggests an underlying skin problem. 2. The patient's symptoms are related to alendazole allergy. It is a medication that causes a person's body to produce antihistamines (medicaments that cause a temporary relief from dizziness or headache) in response to the allergen found in the patient's food and clothing. 3. The treatment for alendazole Allergy involves an antihistamine with broad-spectrum antalgic and antitussive properties, such as pyrantel pamoate. 4. Pyrantel pamoate is a nonsteroidal anti-inflammatory drug (NSAID) that can be used to relieve pain and discomfort from various conditions, including allergies. 5. The patient has previously experienced an allergy to albendazole, which could be a factor for choosing pyrantel pamoate as an alternative medication.   In conclusion, based on the symptoms presented by the patient, their previous allergy to alendazole, and the potential risks associated with using NSAIDs, it is recommended that the patient use pyrantel pamoate as an alternative medication for managing their skin problem related to alendazole Allergy.  **ChatGLM-6B+ Med-RISE**  Step 1: Identify the patient's symptoms  The patient presents with intense perianal itching, particularly at night, and a positive scotch tape test result showing eggs of the parasite Enterobius vermularis.  Step 2: Determine the diagnosis  Based on the patient's symptoms and the positive scotch tape test result, the diagnosis is Enterobius vermularis pinworm infection.  Step 3: List available treatments.  Step 4: Consider the patient's allergy to albendazole  The patient has a documented allergy to albendazole, which makes it a treatment option.  Step 5: Eliminate treatments based on the patient's allergy  Albendazole, mebendazole, and Amphotericin B are eliminated because they are known to cause side effects or are not effective for pinworm infections.  Step 6: Choose the best alternative medication  Ivermectin is a broad-spectrum antiparasitic that is effective against pinworms and can be used as an alternative to albendazole. Pyrantel pamoate is another antiparasitic that is effective against pinworms and could be used as an alternative in the case of albendazole allergy.  Step 7: Inductively reason to select E) Pyrantel pamoate  Pyrantel pamoate is safe and effective for the treatment of pinworm infections and is a suitable alternative for patients who cannot take albendazole or mebendazole due to an allergy.  Therefore, the best alternative medication for the treatment of the patient's symptoms would be E) Pyrantel pamoate. | |
| --- | --- |
| **Pattern A:** Factual Inconsistency  **Pattern B:** Factual Fabrication | **Pattern C:** Instruction Inconsistency  **Pattern D:** Context Inconsistency  **Pattern E:** Logical Inconsistency |

# Figure S2. Example of hallucination types in Chain of Thought (CoT) reasoning in each Large Language Model (Sample 2).

| **MedMCQA [test: 2480]:** Full mouth disinfection is done in:  **A) 24 hours**; B) 48 hours; C) 72 hours; D) 36 hours.  **GPT-Turbo 3.5**  Step 1: Understand the question  The question is asking about the duration of full mouth disinfection.  Step 2: Analyze the options  A) 24 hours  B) 48 hours  C) 72 hours  D) 36 hours  Step 3: Eliminate options based on knowledge or common sense  Full mouth disinfection typically involves a thorough cleaning and disinfection of the entire mouth, including teeth, gums, and oral tissues. This process may take some time to complete.  Option A (24 hours) seems too short for a thorough full mouth disinfection.  Option B (48 hours) and Option C (72 hours) seem more reasonable, as they allow for a longer duration to ensure thorough disinfection.  Option D (36 hours) is closer to Option B (48 hours) and Option C (72 hours), but it may still be too short for a complete full mouth disinfection.  Step 4: Determine the answer  Based on the analysis, the most reasonable option for the duration of full mouth disinfection is Option C) 72 hours.  Therefore, the answer to the question is C) 72 hours.  **GPT-Turbo 3.5 + Med-RISE**  The correct answer to the question of how long full mouth disinfection takes is 'A': 24 hours. Full mouth disinfection is an intensive dental procedure that includes scaling and root planning of all pockets and the treatment of all oral niches. This process is carried out in two visits, all within 24 hours. The goal of this rapid and comprehensive approach is to minimize the chance of reinfection with bacteria from various areas of the oral cavity, including the tongue, tonsils, and interdental spaces.  **GPT-4**  Full mouth disinfection is typically completed within a short time frame such as 24 hours. It is done through one to two sessions of 1 to 1.5 hours each, usually within 24 hours of each other. Therefore, the answer is: A) 24 hours  **GPT-4 + Med-RISE**  Full mouth disinfection, a procedure often used in the treatment of periodontitis, is typically completed in 24 hours (Option A).  24 hours (Option A): This is often considered the optimal time frame. The procedure could be done in one extended session or two sessions within a day. This short period between sessions helps to prevent the repopulation of bacteria in the periodontal pockets.  Other Options (48, 72, 36 hours): Longer intervals between treatment sessions could potentially allow for bacterial repopulation, which might reduce the efficacy of the treatment.  Based on the above considerations, as a doctor, I would lean towards the 24-hour protocol (Option A) for full mouth disinfection. This approach is believed to provide the most effective control of periodontal pathogens by minimizing the window for bacterial re-colonization.  **Vicuna-13B** Step 1: Eliminate options that are not relevant to full mouth disinfection.  Option A: 24 hours is too short a time frame for a full mouth disinfection. This option is not relevant.  Option B: 48 hours is too long a time frame for a full mouth disinfection. This option is not relevant.  Option C: 72 hours is too long a time frame for a full mouth disinfection. This option is not relevant.  Option D: 36 hours is the correct time frame for a full mouth disinfection. This option is relevant.  Step 2: Analyze the remaining option.  Option D: 36 hours is the correct time frame for a full mouth disinfection. This option is relevant.  Therefore, the answer to the question: “Full mouth disinfection is done in: is 36 hours.”  **Vicuna-13B + Med-RISE**  Step 1: What is the aim of full mouth disinfection?  The aim of full mouth disinfection is to complete debridement of all periodontal pocket areas within a short time frame such as 24 hours, in order to minimize the chance of reinfection of the pockets with pathogens coming from other oral niches like the tongue, tonsils, and non-treated periodontal pocket.  **ChatGLM-6B**  Step 1: Identify the question or statement being asked. In this case, the question is “What is the optimal time for full mouth disinfection?”  Step 2: Determine the type of question or statement. In this case, the question is specific to full mouth disinfection.  Step 3: Look for evidence or data to support the answer. In this case, there is no direct evidence or data on the optimal time for full mouth disinfection. However, based on the medical nature of the question, we can infer that the optimal time for full mouth disinfection may depend on factors such as the patient's age, sex, medical history, and other medications being taken.  Step 4: Analyze the options and compare them. There are four options to choose from: 24 hours, 48 hours, 72 hours, and 36 hours. However, given the medical nature of the question, it is important to consider the potential risks and benefits of each option.  Step 5: Consider the potential risks and benefits. Full mouth disinfection can be beneficial in reducing the risk of infection, while also potentially reducing the risk of damage to the mouth and teeth. However, full mouth disinfection can also be time-consuming and may not be practical for all patients.  Based on the information provided, the most appropriate answer is: It is not recommended to perform full mouth disinfection in 36 hours.  **ChatGLM-6B+ Med-RISE**  Based on the medical evidence, we can evaluate the options as follows:  * A) FMD is done in 24 hours. * B) FMD is not done in 48 hours. * C) FMD is not done in 72 hours.  Option A is the most likely answer. Full mouth disinfection is typically done through one to two sessions of 1 to 1.5 hours each, usually within 24 hours of each other. The aim of FMD is to complete debridement of all periodontal pocket areas within a short time frame such as 24 hours, in order to minimize the chance of reinfection of the pockets with pathogens coming from other oral niches like the tongue, tonsils, and non-treated periodontal pocket. This means that FMD is typically completed within 24 hours. Therefore, option A is the most likely answer.  Therefore, option A is the most likely answer. | |
| --- | --- |
| **Pattern A:** Factual Inconsistency  **Pattern B:** Factual Fabrication | **Pattern C:** Instruction Inconsistency  **Pattern D:** Context Inconsistency  **Pattern E:** Logical Inconsistency |
